# A common classification framework for neuroendocrine neoplasms: an International Agency for Research on Cancer (IARC) and World Health Organization (WHO) expert consensus proposal

**DOI:** 10.1038/s41379-018-0110-y

**Published:** 2018-08-23

**Authors:** Guido Rindi, David S. Klimstra, Behnoush Abedi-Ardekani, Sylvia L. Asa, Frederik T. Bosman, Elisabeth Brambilla, Klaus J. Busam, Ronald R. de Krijger, Manfred Dietel, Adel K. El-Naggar, Lynnette Fernandez-Cuesta, Günter Klöppel, W. Glenn McCluggage, Holger Moch, Hiroko Ohgaki, Emad A. Rakha, Nicholas S. Reed, Brian A. Rous, Hironobu Sasano, Aldo Scarpa, Jean-Yves Scoazec, William D. Travis, Giovanni Tallini, Jacqueline Trouillas, J. Han van Krieken, Ian A. Cree

**Affiliations:** 1 Istituto di Anatomia Patologica, Università Cattolica-Fondazione Policlinico Universitario A. Gemelli, Rome, Italy; 2 Department of Pathology, Memorial Sloan Kettering Cancer Center, New York, NY USA; 3 International Agency for Research on Cancer (IARC), World Health Organization (WHO), Lyon, France; 4 University Health Network, University of Toronto, Toronto, ON Canada; 5 University of Lausanne Medical Center, Lausanne, Switzerland; 6 CHUGA, UniversitéUGA, Centre Hospitalier Universitaire Grenoble Alpes, Grenoble, France; 7 Department of Pathology, University Medical Center Utrecht and Princess Máxima Center for Pediatric Oncology, Utrecht, The Netherlands; 8 Charité - University Hospital Berlin, Berlin, Germany; 9 University of Texas MD Anderson Cancer Center, Houston, TX USA; 10 Department of Pathology, Technical University of München, München, Germany; 11 Department of Pathology, Belfast Health and Social Care Trust, Belfast, UK; 12 University Hospital Zurich, Zurich, Switzerland; 13 University of Nottingham, Nottingham, UK; 14 Beatson West of Scotland Cancer Centre, Glasgow, UK; 15 National Cancer Registration and Analysis Service, Fulbourn, UK; 16 Department of Pathology, Tohoku University School of Medicine, Sendai, Japan; 17 Section of Pathology, ARC-Net Research Center and Department of Diagnostics and Public Health, University and Hospital Trust of Verona, Verona, Italy; 18 Departement of Pathology, Gustave Roussy Cancer Campus, Villejuif, France; 19 University Medical Center, University of Bologna, Bologna, Italy; 20 Faculté de Médecine Lyon Est, Lyon, France; 21 Radboud University Nijmegen Medical Center, Nijmegen, The Netherlands

## Abstract

The classification of neuroendocrine neoplasms (NENs) differs between organ systems and currently causes considerable confusion. A uniform classification framework for NENs at any anatomical location may reduce inconsistencies and contradictions among the various systems currently in use. The classification suggested here is intended to allow pathologists and clinicians to manage their patients with NENs consistently, while acknowledging organ-specific differences in classification criteria, tumor biology, and prognostic factors. The classification suggested is based on a consensus conference held at the International Agency for Research on Cancer (IARC) in November 2017 and subsequent discussion with additional experts. The key feature of the new classification is a distinction between differentiated neuroendocrine tumors (NETs), also designated carcinoid tumors in some systems, and poorly differentiated NECs, as they both share common expression of neuroendocrine markers. This dichotomous morphological subdivision into NETs and NECs is supported by genetic evidence at specific anatomic sites as well as clinical, epidemiologic, histologic, and prognostic differences. In many organ systems, NETs are graded as G1, G2, or G3 based on mitotic count and/or Ki-67 labeling index, and/or the presence of necrosis; NECs are considered high grade by definition. We believe this conceptual approach can form the basis for the next generation of NEN classifications and will allow more consistent taxonomy to understand how neoplasms from different organ systems inter-relate clinically and genetically.

## Introduction

The current pathologic classifications of neuroendocrine neoplasms (NENs) across different organ systems use a range of site-specific terminologies and criteria, creating significant confusion among pathologists and treating clinicians. The World Health Organization (WHO) International Agency for Research on Cancer (IARC) has now started the new fifth edition of the WHO Classification of Tumors, published as the widely used WHO Blue Books (http://whobluebooks.iarc.fr). A uniform classification framework for NENs at any anatomical location would reduce inconsistencies and contradictions among the various systems currently in use, allowing unification of classification concepts, despite organ-specific differences in classification criteria, tumor biology, and prognostic factors. The classification suggested here is intended to allow pathologists and clinicians to manage their patients with NENs consistently, and to facilitate comparisons between the different entities falling into this category of neoplasms.

## Methods

A dedicated consensus meeting was held in Lyon on 2–3 November 2017 at IARC, under the auspices of the WHO Classification of Tumors Group. IARC devised the structure, defined the aims, selected the experts and prepared the meeting agenda. Itemized proposal statements and questions were presented, discussed, and consensually agreed upon or discarded by the working group. A resulting “common classification framework” was developed to standardize concepts among NENs of different anatomic sites. Several additional experts were later selected to assist with specific topics. Each subspecialty expert subsequently provided site-specific classification considerations for implementation of the proposed common classification framework.

## Results and discussion

A framework for NEN classification is proposed in which the term NEC is clearly indicative of high-grade malignant histology and biologic behavior. Neuroendocrine tumor (NET), in contrast, is intended to designate a family of well-differentiated neoplasms whose potential to metastasize or invade the adjacent tissues depends on tumor site and type, and grade [1, 2]. In some sites, such as the pituitary and parathyroid, the vast majority exhibit very low risk of metastatic behavior (hence the terminology “adenoma” that has been used in these sites); in others such as the pancreas and small intestine, most NETs behave in a malignant fashion. The difficulty of specifically predicting the behavior of well-differentiated NENs is well-known, and although organ-specific grading schemes have aided in stratifying relative aggressiveness, the proposed conceptual terminology expressly avoids categorizing the neoplasms as explicitly “benign” or “malignant”. Thus, there is no intent for the designation of NET to affect the current understanding of malignant potential, which should remain an organ-specific characteristic. It is hoped that the proposed classification will stimulate interest in exploring potential grading parameters for anatomic sites where there is little information regarding the prognostic significance of grading, and/or where NETs are not currently graded, and to encourage the potential for examining the role of cell proliferation and other grading factors for prognostication in sites where this has not been performed.

### Proposed classification

NENs are relatively rare and comprise a heterogeneous group of tumors characterized by the presence of neurosecretory granules and typically showing a characteristic histology and immunoprofile. Their incidence in the general population varies depending on the specific anatomic location [3, 4]. It was widely acknowledged that:

i. NENs arise at almost any anatomical site, including paraganglia, and are distributed throughout the body in organs of all types, as well as in soft tissues;
ii. NENs at various sites are of epithelial or neuronal/neuroectodermal origin, and share major morphological and protein expression signatures depending on differentiation;
iii. NENs express a variable spectrum of proteins, shared with their normal cell counterparts at specific anatomical locations, including markers of general neuroendocrine differentiation (such as chromogranin A, chromogranin B, and synaptophysin) as well as site-specific markers such as hormones and transcription factors [4].

Existing classification systems vary widely in terminology and criteria between sites, with robust data supporting grading systems in some anatomic sites (e.g., lung, gastrointestinal tract, pancreas), but not in others (e.g., breast, thyroid, parathyroid). In addition, some NETs have been subjected to careful cell-type classification (most well-known in the pituitary, but also in the rectum and pancreas) that has prognostic and predictive value, whereas others have not, e.g., in the female genital tract and breast. The relative prevalence of different NEN categories also varies by anatomic site. The panorama of genetic knowledge regarding NENs is patchy, with well-defined traits defined by high-throughput studies for some anatomic sites and relatively scarce information for other sites.

The term NENs encompasses both well-differentiated NETs and poorly differentiated NECs, as they both share common histologic, immunophenotypic, and ultrastructural neuroendocrine features. However, genetic evidence at specific anatomic sites supports the dual morphological subdivision that distinguishes poorly differentiated NECs from well-differentiated NETs [5–9]. Although they can have overlapping histologic features, their inclusion together in a single classification framework may incorrectly lead to the presumption that well-differentiated NETs and poorly differentiated NECs are closely related neoplasms; in most organs where these families of neoplasms have been studied, the data suggest that they are not biologically closely related [7–9]. In addition, to have different degrees of biological aggressiveness, and different responses to medical therapy, NETs and NECs have different risk factors, hereditary predispositions, relationships to non-NE neoplasia, and underpinning genetics. This is well supported by data in the pulmonary and the digestive systems, as described below, with limited data as yet in other systems.

Six major points of discussion were identified by the expert group: 1. anatomy; 2. tumor category definition; 3. tumor family definition; 4. tumor-type definition; 5. tumor sub-types definition; 6. tumor grading procedures.

1. *Anatomy*: It is recognized that every anatomical site has its own individuality and clinical–pathological features, which often form the basis for historical classification systems. Anatomic site-specific features must be considered when devising any common classification system in order to avoid potential confusion. It was proposed and agreed that current WHO definitions (i.e., site-specific tumor definitions) should be maintained, until potentially revised within the next edition of each WHO Blue Book, and that the novel uniform standard classification terminology for NEN (NEN-WHO 2018) be appended in brackets when it differs from the currently employed site-specific terminology. It was noted that the recently proposed new terminology for pituitary tumors is more in line with this proposal than the current 2017 WHO terminology [10]. Use of this new terminology for pituitary NENs, rather than the 2017 WHO terminology may be helpful to allow for a clear and smooth transition in the classification to assist those using it. We expect that future WHO Blue Books will use the new classification system.
2. *Tumor category definition*: It was proposed and agreed to adopt the term “neuroendocrine neoplasm (NEN)” as a term encompassing all tumor classes with predominant neuroendocrine differentiation, including both well and poorly differentiated forms. Given the multiple anatomic sources (neural structures, endocrine organs and/or neuroendocrine cells), morphology, and the expression of markers of neuroendocrine differentiation (general and specific) were recognized as key features defining these neoplasms at any specific anatomic site. It was acknowledged that the expression of neuroendocrine markers can vary depending on anatomic site and degree of differentiation, and that different general neuroendocrine markers to define neuroendocrine differentiation are currently used in different organ systems (e.g., only chromogranins, and synaptophysin in the gastrointestinal tract and pancreas, versus chromogranins, synaptophysin, and CD56 in the lung).
3. *Tumor family definition*: It was proposed and agreed that two families (or classes) of epithelial NENs be recognized, well-differentiated and poorly differentiated. It was further agreed that classical cytological/histological morphological criteria be adopted for the definition of differentiation (Table 1). It was acknowledged that the two families may not exist in all anatomical sites, and that their relative prevalence also varies widely by site of origin. It was proposed and agreed that the well-differentiated family be designated “neuroendocrine tumor (NET)”, and the poorly differentiated family “neuroendocrine carcinoma (NEC)”. There are some areas of the body where almost all NENs are NETs (e.g., small intestine, ovary, parathyroid, pituitary); in other organs NECs predominate (e.g., lung, colon). As this is primarily a classifier for NEN of epithelial origin, it was further suggested that paragangliomas (i.e., NEN of non-epithelial origin) be regarded as a third family of NENs.

**Table 1:** NEN 2018 WHO proposed classification of selected NEN by site, category, family, and tumor type

Site	Category	Family	Type	Grade	Current terminology
Lung	Neuroendocrine neoplasm (NEN)	Neuroendocrine tumor (NET)	Pulmonary neuroendocrine tumor (NET)^a^	G1 G2	Carcinoid Atypical carcinoid^a^
		Neuroendocrine carcinoma (NEC)	Small cell lung carcinoma (Pulmonary NEC, small cell-type)^b^		Small cell lung carcinoma
			Pulmonary NEC, large cell-type		Large cell NE carcinoma
Uterus (corpus and cervix)	Neuroendocrine neoplasm (NEN)	Neuroendocrine tumor (NET)	Uterine neuroendocrine tumor (NET)	G1 G2 G3	Carcinoid Atypical carcinoid Atypical carcinoid
		Neuroendocrine carcinoma (NEC)	Uterine NEC, small cell-type		Small cell carcinoma
			Uterine NEC, large cell-type		Large cell NE carcinoma
Pancreas	Neuroendocrine neoplasm (NEN)	Neuroendocrine tumor (NET)	Pancreatic neuroendocrine tumor (NET)	G1 G2 G3	PanNET G1 PanNET G2 PanNET G3
		Neuroendocrine carcinoma (NEC)	Pancreatic NEC, small cell-type		Small cell NE carcinoma
			Pancreatic NEC, large cell-type		Large cell NE carcinoma

NEC are regarded as high grade, but as they represent a separate tumor family, there is no need to for formal grading.

^a^The category of G3 atypical carcinoid in the lung is not a validated entity and not recognized in the 2015 WHO classification. Currently such tumors are classified as small cell lung carcinoma (SCLC) or large cell neuroendocrine carcinoma (LCNEC). High-grade NET with features of atypical carcinoid similar to the G3 tumors of the pancreatic/gastrointestinal tract are rare in the lung, not well characterized and need further study.

^b^Not recommended as small cell lung carcinoma (SCLC) is too well ingrained in clinical practice and some SCLC lack commonly used neuroendocrine markers.
4. *Tumor-type definition*: Tumor types (Table 1) represent the diagnostic entities within the families outlined above: for some this is currently the same as the family name with the addition of site (e.g., pancreatic NET), though for others it may differ substantially (e.g., carcinoid tumor, small cell lung cancer). Independent tumor types are recognized by their own ICD-O codes (http://codes.iarc.fr), which should be maintained until revision as part of the WHO Classification of Tumors.
5. *Tumor sub-type definition*: Tumor sub-types (variants) can be defined morphologically or by other criteria, and some may have their own ICD-O codes.
6. *Tumor grade*: It was proposed and agreed that well-differentiated neoplasms (NETs) should usually be graded in three tiers as G1, G2, and G3 (Table 1), corresponding to low-grade, intermediate-grade, and high-grade. In some organs, the current nomenclature inherently reflects the grade (e.g., lung and thymus, where carcinoid tumors are G1 and atypical carcinoid tumors are G2), and therefore current reporting practices do not separately specify the grade. It is not necessary to grade NEC as these are always high grade.
  It was also agreed that three grading parameters of prognostic relevance are:
  i. the mitotic count should usually be expressed as mitoses per mm^2^ area, ideally counted in up to 10 mm^2^ to assure accuracy, unless hotspots are required (e.g., breast). In lung and pancreatic NENs, it is current practice to express the number of mitoses within an area of 2 mm^2^. In practice, tissue availability may restrict areas available for counting. It may also be best practice to specify the number of mitoses counted within the total area assessed for each case (i.e., X mitoses in Y mm^2^);
  ii. the Ki-67 cell labeling index performed on regions of most intense labeling (“hotspots of at least 0.4 mm^2^”) using a validated antibody (i.e., MIB1 antibody) and
  iii. the presence or absence of necrosis, defined by morphological criteria. Necrosis may be focal (punctate) or diffuse (geographic).

Mitotic counts have in the past been expressed as the number per high-powered field (HPF) as the unit of area within the tumor. Unfortunately, different combinations of microscopes and lenses result in HPFs of variable area [11–13]. Grade may therefore differ, simply based on the microscope being used. While it is possible to at least define the exact size of these fields in scientific publications, this does not allow an accurate grade to be assigned in routine practice. It is arguable that there is little excuse for the use of HPFs, when international standard (SI) units such as mm^2^ are available, and we have chosen to express the mitotic count per mm^2^, in line with WHO Blue Book policy.

It was agreed that the specific basis for grading should continue to be contingent on anatomic site, based on current practices for each site. Mitotic count and/or Ki-67 labeling index are the minimum required for grading at almost any anatomic site (where grading is mandated). It was proposed and agreed that poorly differentiated neoplasms NEC be (i) of high grade by definition; (ii) of two separate morphologic types and (iii) defined as small cell neuroendocrine carcinoma (SCNEC) or large cell neuroendocrine carcinoma (LCNEC). Some tumor types may have organ-specific names: e.g., small cell lung cancer (SCLC), although small cell carcinoma should not be abbreviated to SCC to avoid confusion with squamous cell carcinoma (SCC). It was proposed and agreed that tumor classes be site-specific and different, and site-specific grading parameters (cut-offs) be defined for each anatomic subgroup.

It was proposed and agreed that in the pathology report: (i) the parameters used for grading (mitotic count, Ki-67 labeling index [%] and necrosis) be stated clearly; (ii) the site-specific tumor nomenclature according to current WHO classifications be stated first; and (iii) the novel uniform standard classification framework be added in brackets, i.e., (NEN-WHO 2018).

### Additional points

NENs in some anatomic sites are further characterized based on their production of bioactive substances (peptide hormones or bioamines), and in a number of anatomic sites, clinically functional NENs exist in which a hormonal or paraneoplastic syndrome may be the dominant clinical manifestation of the neoplasm. It was acknowledged that the detection of secretory products, either in the serum or using immunohistochemistry to assay the tumor cells, may be of relevance for classification (i.e., in the pituitary), for prognosis (such as in pancreatic insulinomas), or to correlate with the clinical symptoms in selected patient populations. However, given the variety of different bioactive substances produced in NENs of different locations, no general recommendations for assaying them could be developed.

In many anatomic sites, neoplasms exist that exhibit both neuroendocrine and non-neuroendocrine elements, which can be present in morphologically distinct cell populations or more intimately intermixed. The neuroendocrine elements of these “mixed” or “combined” neoplasms are most commonly NECs [14–16]; the non-neuroendocrine components can be glandular, squamous, or other lineages. Designations such as combined small cell carcinoma (in the lung) mixed adenoneuroendocrine carcinoma (MANEC; in the tubular gastrointestinal tract), or mixed neuroendocrine-non-neuroendocrine neoplasm (MiNEN; in the pancreas) have been proposed for this family. While this conceptual category is recognized as an important member of the NEN family, these complex neoplasms were not included in the present classification framework, though they are mentioned in the site-specific sections below where they may be a cause of confusion.

Another scenario in which neuroendocrine differentiation can occur in neoplasms is in non-NECs following chemotherapy, molecularly targeted therapy, or radiotherapy. In some instances, a small cell carcinoma may arise following treatment of an adenocarcinoma (such as in the prostate or lung) and such poorly differentiated NECs can be considered within the present classification framework. In other scenarios, however, treated carcinomas may display apparent well-differentiated neuroendocrine elements, such as in the Paneth-like cell features of treated prostatic adenocarcinoma [17, 18], or the well-differentiated neuroendocrine cell nests in rectal carcinomas following chemoradiotherapy [19].

Finally, tumors of the paraganglia are designated paraganglioma and are classified based on criteria for these neoplasms: they are mentioned in passing, but are not the focus of this paper.

## Implications for site-specific classification

**Fig. 1:**
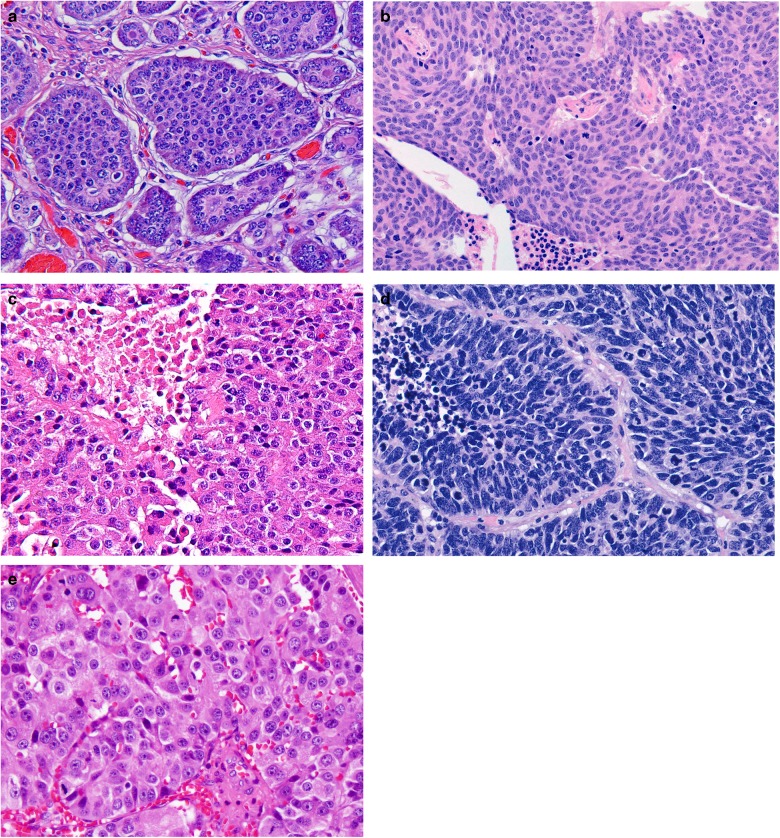
The H&E appearance of NEN from different sites: **a** a grade 1 NET from the ileum, **b** a grade 2 NET from the lung (atypical carcinoid), **c** a grade 3 NET from the pancreas, **d** a NEC (SCLC) from the lung, and **e** large cell NEC from the pancreas

The above classification framework criteria (Fig. 1) was proposed and agreed to be applied to each anatomical site in which NENs arise. It was recognized that NENs at different anatomic sites may fit variably into the above-defined framework. Accordingly, the site-specific applications for the classification are further defined below.

### Pancreatic and gastrointestinal tract NENs

**Fig. 2:**
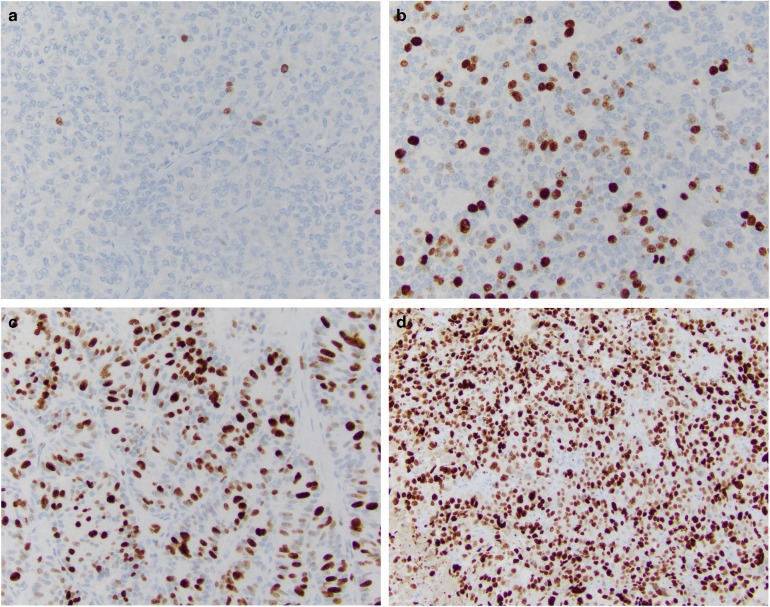
Ki-67 staining of NEN from different sites: **a** a grade 1 NET from the ileum, **b** a grade 2 NET from the lung (atypical carcinoid), **c** a grade 3 NET from the pancreas, and **d** a NEC (SCLC) from the lung

The current proposed NEN classification (Table 1) is largely based on the recently updated WHO classification for pancreatic NENs [16]. This classification separately distinguishes pancreatic well-differentiated NE tumors (PanNETs) and poorly differentiated NE carcinomas (PanNECs) morphologically [1, 2, 20–22]. Grading of PanNETs into three tiers (G1, G2, and G3) is based on proliferation assessed by mitotic count and Ki-67 index. Necrosis, though recognized as a potential adverse prognostic factor, is not included in the grading parameters. In the 2017 WHO classification, PanNECs are also designated as G3, whereas in the current proposal NECs are not specifically graded, as they are regarded all to be high grade by definition. In the pancreas, high-grade NENs are uncommon, and it appears that G3 PanNETs are at least as frequent as PanNECs, in contrast to the gastrointestinal tract (see below) (Fig. 2).

In the pancreas, NETs display recurrent somatic mutations in *MEN1*, *DAXX*, *ATRX*, *PTEN*, and members of the mTOR signaling pathway [23–25]. Clinically, sporadic NETs also present germline mutations in the DNA repair genes *MUTYH*, *CHEK2*, and *BRCA2* [24]. In contrast, NECs instead commonly have mutations in *TP53* and *RB1* and may share mutations in *KRAS* and *SMAD4*, genes commonly involved in the pathogenesis of ductal adenocarcinoma [26–28]. Pancreatic neuroendocrine carcinomas (PanNECs) are usually large cell-type and may contain components of adenocarcinoma, typically not found in NETs. Progression from G1 or G2 NETs to G3 may occur, both within a primary tumor and between sites of disease, particularly over time as the tumor evolves clinically. Very rare, conversely, is the progression from G3 NET to NEC—if it occurs at all: further evidence is required. As in pancreatic NETs (PanNETs), gastrointestinal NETs (GI-NETs) are mutationally quiet, with the most frequent mutated gene being *CDNK1B*, which harbor mutations in 8% of small intestine NETs [26, 29]. In the gastrointestinal tract, G3 NETs are also reported, though less commonly than in the pancreas. SSTR2A expression is usually recognized in PanNETs, while it is only occasionally observed in PanNECs [30].

Clinical data demonstrate the dependency of prognosis on grade, with G2 PanNETs being more aggressive than G1. G3 PanNETs also appear to be somewhat more aggressive than G1 or G2, but they are not as aggressive as PanNECs, which are rapidly lethal in most cases. As in other anatomic locations, PanNECs are felt to respond best to platinum-based chemotherapy, whereas PanNETs are more optimally treated with somatostatin analogs, mTOR inhibitors, alkylating agents, or VEGF inhibitors [31–33]; these differences in clinical management also emphasize the importance of distinguishing PanNETs from PanNECs. The value of determining the hormone secretion profile of pancreatic NENs is debated, although insulinomas are usually less aggressive, and gastrinomas more so.

In the gastrointestinal tract, the classification of NENs has not been updated by WHO since 2010 [14], though this is now in progress. At that time, NET G3 was not a recognized category, and all G3 NENs were regarded to be poorly differentiated NECs; hence, the current classification differs in that regard from the present proposal. In fact, G3 NETs in the gastrointestinal tract have also been reported [25], although less commonly than in the pancreas. Therefore, it has been proposed that a three tier system (G1–G3) should be adopted for NETs in the gastrointestinal tract as well [2]. However, most high-grade NENs of the gastrointestinal tract are NECs, with mutations in *TP53* and *RB1* and, in the colon, *APC* mutations similar to those found in adenocarcinomas, which are not usually reported in NETs [27, 28]. As in PanNETs, there is a low overall incidence of mutations in gastrointestinal NETs; for example, small bowel NETs have an 8% incidence of *CDNK1B* mutations and few other recurrent mutations [26, 34]. Instead, epigenetic dysregulation appears to have a major role in the pathogenesis of small bowel NETs [35]. NECs of the gastrointestinal tract may exhibit components of adenocarcinoma or, in the esophagus or anus, squamous cell carcinoma, again emphasizing the close relationship of NECs to non-NECs.

### Lung NENs

In the lung NENs are currently classified as low-grade typical carcinoid, intermediate-grade atypical carcinoid, and the high-grade LCNEC and small cell lung carcinoma (SCLC) [15, 36, 37]. Use of this terminology and the 2015 WHO criteria were recommended by a recent ENETS guideline based upon a systematic literature review and consensus of an international, multidisciplinary panel of experts and endorsed by the International Association for the Study of Lung Cancer [38]. typical carcinoid and atypical carcinoid are well-differentiated and correspond to NET, while LCNEC and SCLC are poorly differentiated and correspond to NEC within the classification proposed here (Table 1). Up to 25% of surgically resected SCLC and LCNEC have histologic components of other non-small cell carcinomas such as adenocarcinoma or squamous cell carcinoma and these tumors are classified as combined SCLC or combined LCNEC, respectively [36, 37]. In contrast to SCLC and LCNEC, carcinoids characteristically do not have components of non-small cell carcinoma.

Since the 1999 and 2004 WHO classifications [39, 40] these tumors have primarily been distinguished based on mitotic counts per 2 mm^2^, the presence or absence of necrosis and for the high-grade NEC, whether the tumor has small cell or large cell cytologic features [15].

The main role of Ki-67 in lung NENs is to distinguish the carcinoids from the high-grade LCNEC and SCLC. This is particularly important in small biopsies with crush artifact, where carcinoids can be misdiagnosed as SCLC [41, 42]. No reliable cut-off has been established for Ki-67 in the distinction between typical carcinoid and atypical carcinoid [43], although reported ranges for typical carcinoid are 2.3 to 4.15% and for atypical carcinoid are 9 to 17.8% [43]. Although some studies have suggested usefulness of Ki-67 in grading lung carcinoids, others have shown limited additive value over histologic criteria, particularly mitotic counts [44, 45]. In addition there is no well-defined threshold to distinguish carcinoids from SCLC or LCNEC, however a wide range of cut-off values from 2.5 to 30% have been proposed. Some studies have evaluated the entire spectrum of neuroendocrine lung neoplasms with various proposals of how to incorporate Ki-67 proliferation rates and mitotic counts, but there is no consensus on the optimal approach [38, 43, 46, 47]. There is a great need for further research on this topic both on the issue of separating typical carcinoid from atypical carcinoid and carcinoids from the high-grade LCNEC and SCLC.

The category of G3 atypical carcinoid in the lung is not a validated entity and not recognized in the 2015 WHO classification, although a few studies suggest these cases may exist [48, 49]. Currently such tumors are classified as SCLC or LCNEC. High-grade NET with features of atypical carcinoid similar to the G3 tumors of the pancreatic/gastrointestinal tract are rare in the lung, not well characterized and need further clinical, pathologic and genetic evaluation [48, 49].

Within the lung, 95% of NENs are high grade poorly differentiated, including SCLC (79%) and LCNEC (16%) with the carcinoids only comprising ~5% (5% typical carcinoid and 0.5% atypical carcinoid) [50, 51]. Poorly differentiated NECs typically present in older patients, with strong association with cigarette smoking and a very poor prognosis. Clinically, SCLC is distinct from all other non-small cell lung cancers and the other NENs in that it consistently shows an initial clinical response to cisplatin/etoposide chemotherapy. Responsiveness to SCLC chemotherapy regimens has been reported in some LCNEC series [52], but this is not a consistent finding [37, 52]. Typical carcinoid and atypical carcinoid occur in younger patients than SCLC or LCNEC and they do not show a strong association with cigarette smoking [50]. Current evidence suggests these tumors show less benefit from traditional platinum-based chemotherapy, however everolimus, an MTOR pathway inhibitor, is now approved, and recent evidence shows temozolamide may have some effect.

Global genomic studies have demonstrated extensive genetic alterations in SCLC and large cell carcinoma (including LCNEC), consisting of amplifications, deletions, and mutations in contrast to very few genetic changes in lung carcinoids [53]. SCLCs characteristically have biallelic inactivation of *TP53* and *RB1* [54–56]. In addition, SCLCs show inactivating mutations in NOTCH family genes in 25% of cases and in rare cases kinase gene mutations [57]. Several studies have shown that LCNECs are more genomically heterogeneous than SCLCs, with a group that is similar to SCLC, with biallelic inactivation of *TP53* and *RB1*, and another group that is more non-small cell-like, with mutations in *KRAS*, *STK11/KEAP1* [48, 57]. In contrast, lung carcinoids lack mutations in *TP53, RB1, KRAS, STK11/KEAP1*, but show frequent mutations in chromatin-remodeling genes such as covalent histone modifiers in 40% and subunits of the SWI/SNF complex including the *MEN1, PSIP1,* and *ARID1A* genes in 22% of cases [58, 59]. Rare cases of LCNEC with carcinoid-like genetic features such as *MEN-1* mutations have been reported [48]. These data demonstrate that, although pulmonary NETs (typical carcinoid and atypical carcinoid) are regarded as part of the spectrum of pulmonary NE neoplasms, they are only distantly related to poorly differentiated NECs (SCLC and LCNEC) because these groups of tumors have major clinical, epidemiologic, histologic, genetic, and prognostic differences.

### Pituitary

The current WHO classification consists only of well-differentiated neoplasms classified as adenomas or well-differentiated carcinomas based on the presence of distant metastasis and sub-classified depending on hormone production. However, despite the rarity of distant metastatic spread, these tumors are recognized to have a high incidence of invasion of surrounding tissues. As recently defined, “aggressive tumors with invasion, and unusually rapid tumor growth, multiple recurrences despite optimal therapies” cause significant morbidity and mortality [60]. Clinically and pathologically, these aggressive tumors and carcinomas with metastasis were very similar [61–63]. It is exceptionally unusual for a pituitary carcinoma to present with synchronous metastasis; they usually develop metachronously, usually after the initial presentation with an adenoma, leading to the awkward situation where a tumor is classified as “adenoma”, and then must be reclassified as “carcinoma” when it spreads. Therefore, it has been proposed that pituitary tumors be classified as NETs, i.e., pituitary NETs (PitNETs) rather than adenomas or carcinomas [10]. Poorly differentiated NECs do not occur in the pituitary.

PitNET prognosis and prediction relies more on cell type and degree of cell differentiation than on proliferative markers [10]. Indeed, this organ has been so well scrutinized that “poorly differentiated tumors” are currently defined based on expression of transcription factors with loss of differentiated cell morphology and hormone production [64, 65], but these tumors remain well-differentiated NETs based on criteria applied in other sites. Although mitotic count and/or Ki-67 index are not useful in clinical practice, it has been recently shown that these proliferative markers have a major impact on PitNET prognosis [66], while others have not [67]. However, grading of these tumors as G1, G2, and G3 is currently not possible based on available data. Mitoses are uncommon in these tumors and there are no data on the value of mitotic counts in the classification of PitNETs. Necrosis is rare and related to vascular thrombosis.

There is some evidence that different mutations underlie tumors of different cell types. For example, *GNAS* mutations may be implicated in the pathogenesis of densely granulated somatotroph/mammosomatotroph tumors, and *USP8* mutations in densely granulated corticotroph tumors. In contrast, *AIP* mutations may be implicated in some sparsely granulated somatotroph tumors with epigenetic silencing in those tumors without mutation. Interestingly, *MEN1* mutation in PitNETs is not specific to the cell type. Early studies suggested that TP53 inactivation and *RAS* mutations were features of carcinomas [68, 69]. However, the genetic factors underlying the majority of sporadic PitNETS remain unknown and epigenetic alterations are thought to be common.

Paragangliomas arising in and around the sella turcica should be distinguished from PitNETs and classified as a separate family.

### Head and neck, including thyroid and parathyroid

#### Nasal cavity, larynx, trachea NEN, neck and parotid gland

Epithelial neuroendocrine neoplasms in the 2016 Head and Neck WHO tumor blue book are categorized into well-differentiated (typical carcinoid), moderately differentiated (atypical carcinoid) and poorly differentiated (small and large cell) neuroendocrine carcinomas. In the proposed nomenclature, based on a recent proposal, they are collectively termed NENs (Table 1) [70] with well-differentiated (typical carcinoid) and moderately differentiated (atypical carcinoid) carcinomas are defined as NETs, grades 1 and 2, respectively and the poorly differentiated neuroendocrine carcinomas as NECs; SCNEC and LCNEC. Well-differentiated NEN (typical carcinoid), NET-G1, display organoid formation composed of monotonous cells with minimal mitotic figures [71]. The differential diagnoses include paraganglioma and medullary thyroid carcinoma [72–74]. Moderately differentiated (atypical carcinoid), NET-G2, carcinoma retains organoid architecture and manifest cellular pleomorphism, moderate numbers of mitoses, and occasional necrosis and amyloid-like deposition. Although not widely practiced, Ki-67 scoring can be helpful. The main differential diagnoses are medullary thyroid carcinoma and paraganglioma [75–77].

In the nasal cavity and paranasal sinuses, the most common NENs are poorly differentiated SCNECs. The differential diagnosis of this entity is broad and includes neuroblastoma, embryonal rhabdomyosarcoma, sinonasal undifferentiated, NUT carcinoma, pituitary NETs, paraganglioma, mucosal melanoma, and primitive neuroectodermal tumors. Lineage-associated immunohistochemical markers are needed in the diagnosis and categorization of these entities. Undifferentiated sinonasal carcinomas do not express neuroendocrine markers, while paraganglioma, neuroblastoma, rhabdomyosarcoma and melanoma are keratin negative. NUT carcinoma is negative for neuroendocrine markers and positive for nuclear protein in testis (NUT-M1 antibody). Primitive neuroectodermal tumor is positive for CD99 and FLI-1 protein. In the neck nodes and parotid gland, NENs comprise of poorly differentiated SCNEC and Merkel cell carcinoma (MCC). MCC commonly express dot-like CK20 staining [78]. Metastasis from skin and other sites must be excluded.

#### Thyroid NENs

The vast majority of thyroid NENs are tumors of C-cells (parafollicular cells), traditionally known as “medullary thyroid carcinoma” (MTC) [79]. MTCs currently represent 3–5% of all thyroid carcinomas and develop in the setting of MEN2 syndromes in ~30% of the cases [80]. Most MTCs are well-differentiated NETs, based on the expression of calcitonin and TTF-1; aggressive, poorly differentiated NECs represent less than 1% of MTCs, and include a small cell variant that needs to be distinguished from small cell carcinoma metastatic to the thyroid gland, especially since small cell carcinomas of many anatomic sites express TTF-1 [81, 82]. The prognosis of MTC is heavily influenced by the stage of the disease, by serum calcitonin and carcinoembryonic antigen levels, and in MEN2 cases by the type of RET mutation [80]. RET mutations influence the tumor microenvironment and angiogenesis, and among sporadic cases p.M918T RET has been linked to poor prognosis, compared to MTCs that are RAS mutated or without mutations [77, 83, 84]. The Ki-67 labeling index in MTC is often <1% (and therefore difficult to assess); nevertheless, limited evidence indicates that Ki-67-based grading may be of prognostic significance [85, 86]. Mitotic count, necrosis and/or Ki-67 labeling index may be used as markers of aggressive behavior but they are not currently part of any validated grading system. Interestingly, however, there is evidence that immunohistochemical loss of calcitonin expression with retention of CEA is considered an unfavorable sign, pointing to the potential value of biomarkers, including hormones, in defining MTC prognosis [75]. Improved biomarker and grade profiling offers a great opportunity for the optimal selection of patients to be treated with the tyrosine kinase inhibitors (Vandetanib, Cabozantinib) currently approved for advanced MTC [80].

Mixed medullary and follicular cell carcinomas, where neoplastic C-cells are closely intermixed with other types of non-neuroendocrine follicular cell-derived tumors (usually papillary carcinoma), are extremely rare but well documented. They correspond to the mixed neuroendocrine-non-neuroendocrine tumors of other organs (e.g., pancreatic mixed neuroendocrine neoplasms), and need to be distinguished from collision MTC-follicular cell-derived tumors and from the rare amphicrine MTC variant where cytoplasmic mucin accumulates within neoplastic C-cells [76]. Equally rare are intrathyroidal NEN with the features of paraganglioma, that need to be distinguished from the paraganglioma-like MTC variant [87].

#### Parathyroid NENs

The current classification includes well-differentiated neoplasms classified as adenomas, atypical adenomas, or carcinomas (parathyroid NETs); poorly differentiated, aggressive carcinomas corresponding to parathyroid NECs are extremely unusual. The diagnosis of malignancy is based on invasive growth, evidenced by vascular invasion, full penetration of the tumor capsule with extension into the surrounding non-neoplastic tissues, or metastases [88, 89]. Mitoses, atypical mitoses, macronucleoli, thick intersecting fibrous bands, and necrosis are potential signs of malignancy [89, 90]. The Ki-67 labeling index is often >5% in carcinomas compared with adenomas and hyperplastic nodules, but there is a significant overlap in individual equivocal cases [91]. Therefore, although the Ki-67 labeling index, mitotic counts, and necrosis are often used as markers of aggressive behavior, they are not part of a formally defined diagnostic grading scheme.

The parafibromin gene (CDC73, previously HRPT2) is frequently inactivated in malignant tumors, and loss of function mutations are identified in the germline of patients with apparently sporadic parathyroid carcinoma (as well as in other CDC73 related disorders, such as hyperparathyroidism-jaw tumor syndrome and familial isolated hyperparathyroidism). Lack of immunohistochemical expression of parafibromin combined with immunoreactivity for PGP9.5 provides a useful diagnostic adjunct to the diagnosis of carcinoma [92]. Parathyroid NENs are a well-known component of MEN1, MEN2A, and MEN4 syndromes, although *MEN1* gene inactivation is not associated with malignant behavior. A variety of genetic alterations including *CCND1* amplification, alterations of the PI3K/AKT/mTOR pathway and overexpression of *CCND1* (previously *PRAD1*) have been identified by high-throughput genetic screening in parathyroid carcinoma and adenoma [93, 94].

### Breast NENs

NENs of the breast are rare and poorly defined. Apart from rare cases of small cell carcinoma, analogous to its pulmonary counterpart, the definition of NENs in the breast varies widely, resulting in variable incidence from <0.1% [95] up to 20% [96]. Most are likely to represent mixed NENs. Clinical syndromes related to hormone production are extremely rare in breast NENs and the classic organoid features of carcinoid tumors of the lung and gastrointestinal tract (i.e., ribbons, cords, and rosettes) are not features of primary NENs of the breast [97]. The 2012 WHO Working Group included NENs under the category “carcinomas with NE features” and defined these as tumors exhibiting morphological features similar to those of NE tumor of gastrointestinal tract and lung and expressing NE markers (i.e., chromogranins, and synaptophysin) to any extent [97]. They classified NENs in the breast into (1) NETs, well-differentiated; these include low and intermediate-grade tumors, which by definition in the breast are malignant, and based on the presence of a peripheral myoepithelial cell layer they are classified and managed as either in situ or invasive disease; (2) NECs, poorly differentiated/small cell carcinomas; these neoplasms, based on the description, included SCNEC but not LCNEC [97]. The current classification also acknowledged the presence of a third category which comprises a subset of breast carcinomas with neuroendocrine differentiation as determined by histochemical and immunohistochemical analysis. These include breast carcinoma of no special type (NST) as well as special types such as solid papillary carcinoma and the hypercellular variant of mucinous carcinoma of any histological grade. Therefore, distinction between well-differentiated NETs and grade 1 or 2 breast carcinomas expressing neuroendocrine markers should be based on the presence of histological features characteristic of neuroendocrine differentiation. Presence of ductal carcionoma in situ (DCIS), estrogen receptor expression (which is present in almost all well-differentiated NETs and in more than 50% of poorly differentiated NECs), axillary node metastasis, and lack of a history of an extramammary primary NEN can support that a breast NEN is primary in that location. Assessment of prognostic variables including mitotic count and Ki-67 labeling index is used as a marker of aggressive behavior, although in a way similar to other breast carcinomas and not as a formally defined grading system for NENs. Unlike most other sites, necrosis is not used as a well-established prognostic factor in NENs of the breast [97]. Tumor stage and histological grade, which encompass mitotic counts, are used as the main prognostic parameters. Currently, there are no data from prospective clinical trials on optimal management of NENs of the breast and these tumors are usually treated with the same strategy used for the other types of invasive breast cancer. Thus, outside of the context of the exceedingly rare small cell carcinoma of the breast, neuroendocrine differentiation in breast neoplasms is not regarded to have therapeutic significance.

### Genito-urinary system and male and female genital organ NENs

#### Genito-urinary system and male genital organs

The 2016 WHO classification of Tumors of the Urinary System and Male Genital Organs [98] introduced a novel terminology for NETs, with well-differentiated NETs, LCNEC, SCNEC, and paraganglioma for NENs of the kidney, prostate, and bladder. The terms carcinoid, typical and atypical carcinoid are not recommended. The classification in all locations is based on morphology but proliferation markers as well as necrosis are not formally included in the classification parameters.

NETs in the kidney, formerly designated carcinoid tumors, are extremely rare, and high-grade NENs arising from the renal pelvic mucosa must be excluded before the diagnosis of NEN of renal parenchyma because they are more common than tumors of renal origin [98]. Up to 15% of well-differentiated NETs arise in a horseshoe kidney [99, 100]. In cases of renal paraganglioma, tumors arising from the perihilar sympathetic ganglia must also be excluded. Some studies have correlated poor patient prognosis with increased mitotic activity, presence of necrosis and cytological atypia, but stage at presentation is the strongest predictor of survival. Poorly differentiated NECs (small cell and large cell types) are aggressive with most patients dying of metastasis.

NETs and NECs of the bladder are derived from the urothelium. For a tumor to be classified as NEC, the typical neuroendocrine histology must constitute the majority of the tumor. Some reported cases were associated with a lesser component of conventional urothelial carcinomas. NETs of the bladder are extremely rare and present as small polypoid masses (mean diameter 5 mm).

Most acinar prostate adenocarcinomas demonstrate scattered neuroendocrine cells by immunohistochemistry. True well-differentiated NETs and LCNEC of the prostate are exceptionally rare. Prostatic NETs must be distinguished from prostatic adenocarcinomas showing extensive Paneth-like differentiation, which can be present initially or following androgen deprivation therapy [101]. SCNECs are frequently mixed with prostate acinar adenocarcinomas or have a history of usual prostatic adenocarcinoma in 40–50%. It is therefore thought that SCNECs represent transdifferentiation from usual prostate adenocarcinoma.

#### Female genital organs

NENs are uncommon or rare at all sites in the female genital tract. They are most common in the ovary where most are clinically benign, morphologically corresponding to carcinoid tumors, and arise in dermoid cysts. The uterine cervix is the most common site of NECs in the female genital tract. The terminology has been confusing in the past, and to some extent currently, due to different nomenclatures being used at different sites. The updated 2014 WHO Classification [102] introduced changes to the terminology of NENs at most, but unfortunately not all, sites in the female genital tract [103]. In the uterine cervix and corpus and vulva, categories of low-grade NET and high-grade NEC are used in WHO 2014 [102], which correspond to NET and NEC in the currently proposed system; the vulva also includes the category of MCC. These terms replace the various categories of carcinoid tumor, atypical carcinoid and small cell and large cell NEC used in WHO 2003 [104]. In WHO 2014 [102], carcinoid and atypical carcinoid are considered as synonymous with low-grade NET while small cell and large cell NEC are synonymous with high-grade NEC. The classification is based on morphology, and mitotic count; proliferation markers and necrosis are not formally included in the classification parameters.

In the 2014 WHO classification of ovarian tumors, there is no separate category of NENs, unlike at other sites in the female genital tract [103]. This is a shortcoming of the 2014 classification. In the ovary, the NEN types included in WHO 2014 [102] are: (1) carcinoid tumor (sub-types of strumal and mucinous carcinoid), which is included in the category of monodermal teratoma and somatic-type tumors arising from a dermoid cyst; (2) small cell carcinoma, pulmonary type; the latter is essentially a SCNEC which is included in the category of miscellaneous tumors and must be distinguished from ovarian small cell carcinoma of hypercalcaemic type, a non-NEN associated with mutations in SMARCA4 [105], and (3) paraganglioma which is included in the category of miscellaneous tumors. There is no category in the WHO 2014 ovarian classification covering the entity of LCNEC.

### Adrenal gland and paraganglia

Tumors deriving from the neurectoderm of the neural crest can occur throughout the body, from the sellar region to the rectum and cauda equina, and may thus represent a diagnostic challenge. When located in the adrenal medulla they are called pheochromocytomas and in all other locations they are called paragangliomas. With regard to the proposed classification framework, all of these neoplasms are regarded to be well-differentiated and therefore NETs; poorly differentiated NECs do not occur in the adrenal or in paraganglia. Because of their embryologic origin, these neoplasms are distinct from other NENs in that they are not epithelial and thus do not express keratins, which may help distinguish them from epithelial NENs. In contrast, they express the transcription factor GATA-3 and often display a population of S100 protein-positive sustentacular cells that surround the nests of tumor cells. Tumors of the adrenal cortex and neuroblastic tumors, arising from the adrenal medulla in infants and children, are beyond the scope of this paper.

Pheochromocytomas and paragangliomas are histologically very similar, having a nested growth pattern (the so-called “Zellballen” pattern) composed of cells with ample granular cytoplasm. Nuclear atypia may sometimes be present or even striking. The most striking difference between pheochromocytomas and paragangliomas is the cytoplasmic staining, which is usually more basophilic in pheochromocytomas and more eosinophilic in paragangliomas. The clinical course of pheochromocytomas and paragangliomas is variable, with the majority of patients being cured by surgery. In a minority of cases, metastases occur. This has led to the notion that all pheochromocytomas and paragangliomas should be considered potentially malignant. Many attempts have been made to predict the behavior of pheochromocytomas and paragangliomas. These attempted grading systems have used histological characteristics and in addition, biochemical or immunohistochemical criteria. In the recent new edition of the WHO volume on endocrine tumors [16], it was concluded that there is no wide acceptance of any grading system and that some systems were awaiting independent confirmation. The two most recent and most promising grading systems are those by Thompson and by Kimura. Thompson proposed a pheochromocytoma of the adrenal gland scaled score (PASS) based on 12 histological criteria (8 criteria scoring 2 points and 4 criteria scoring 1 point) for a total score of 20 [106]. A compound score of 4 or more would indicate adverse clinical behavior of the tumor. Kimura proposed a grading system for adrenal pheochromocytoma and paraganglioma (GAPP) based on 4 histological characteristics, the Ki-67 labeling index, and biochemistry (catecholamine secretion pattern) [107]. This was used to create a three-tiered grading into well-differentiated, moderately differentiated and poorly differentiated pheochromocytoma or paraganglioma, that correlated with statistically significant 5-year and 10-year survival differences. It should be noted that well, moderately, and poorly differentiated categories would correspond to low, intermediate, and high-grade categories in the proposed classification framework. SDHB immunohistochemistry potentially had additional value in predicting metastasis. Specifically, for Ki-67 labeling index, cut-offs of <1%, 1–3%, and >3% were used, based on counting 500–2000 cells in two of the most highly labeled areas, selected by eyeballing. The value of these scoring systems remains unclear and they await widespread application.

### Skin NENs

The prototypical primary cutaneous NEN is the so-called MCC. Its etiology is related to the clonal integration of the Merkel cell polyomavirus and/or ultraviolet radiation [108]. MCC is a high grade, poorly differentiated neoplasm that would be categorized with the NECs in the proposed classification framework. Neither proliferation parameters nor the presence of necrosis are formally needed for its status as a high-grade carcinoma. The main prognostic factor is tumor size. The spectrum of MCC includes small cell, intermediate-size, and large cell cytology, so the general diagnosis of MCC does not rigidly conform to the dichotomous separation of small cell carcinoma and large cell NEC, within the NEC group. Furthermore, a significant differential diagnosis with MCC is pulmonary type small cell carcinoma, and a variety of studies have emphasized the distinguishing histologic and immunophenotypic features of these two entities (11175640; 21453956) [109, 110]. Rare primary cutaneous large cell NECs other than MCC have been reported, but they likely represent sweat gland carcinomas with neuroendocrine differentiation [111].

Most carcinoid or atypical carcinoid-like low or intermediate-grade NETs found in the skin are metastatic lesions [112]. While there are a few case reports of primary cutaneous well-differentiated, low-grade NENs [113], most of them are best classified as low-grade sweat gland carcinomas with neuroendocrine differentiation displaying immunoreactivity for chromogranins, and/or synaptophysin. Some alleged NETs may be sebaceous neoplasms with a carcinoid-like pattern or basal cell carcinomas partly expressing chromogranin A. While a true primary cutaneous well-differentiated NET is not impossible, it is at best exceedingly rare, precluding the need for a grading system.

## The future

The uniform classification framework we have proposed for the universal classification of NENs is based on the common morphology that these neoplasms display at different anatomic sites. It is reasonable to expect that such morphology is the result of a common “neuroendocrine” multigene program functioning at all anatomic sites and driving the neuroendocrine cell commitment. Despite the common morphology, NENs speak different clinicopathological languages depending on their site of origin. Along the same lines, it is expected that tissue-specific neuroendocrine differentiation programs act at specific anatomic sites, deciding the neuroendocrine cell fate and dictating tissue-specific hormonal production. It is also likely (and for the stomach it is proven) that this programming may respond to specific local and general physiologic or pathologic stimuli. Nevertheless, there are situations where NENs express hormones ectopically, emphasizing the relationships between tumors from different sites, and sometimes a tumor presents as a metastatic focus with no known primary, and so it is important to be able to classify such lesions in a rational consistent way irrespective of the site of origin. Our classification framework, although based on solid morphological grounds, lacks an equally solid genetic basis across all anatomic sites. The major hope we have and wish to foresee in the future is that such programs will be unveiled by the massive genetic analyses that are now possible. The genetic landscapes of lung, pancreas and small intestinal NENs have been recently published [6–9, 48, 59] and lay the groundwork for similar studies in other organs. A universal analytic approach of available NEN databases is very much required and we identified a number of other important research needs (Box 1).

### Box 1 Some research needs identified by the expert group to illustrate the studies required to advance understanding of NEN

- General: Further genetic studies of NEN are required in many sites, ideally with computational pathology and phenotypic data on outcome. What are the common genetic and genomic features (and the differences) of NEN from different organs?
- General: Computational pathology studies of Ki-67 proliferation, and mitotic count per mm^2^, are required to assess whether grade is a continuous or categorical variable, including validation against microscope counting (including inter-laboratory and observer reproducibility studies). What thresholds should be applied in clinical practice to separate grades?
- General: What is the prevalence and clinical significance of tumor heterogeneity for mitotic counts and K-67 proliferation index in NEN?
- General: Do NET and NEC occur in all anatomical sites?
- General: What are the distinguishing genetic features of NEC and NET?
- General: What is the nature of mixed neuroendocrine:non-NETs of all organs?
- General: Coordination of NEN databases is required to allow ease of data comparison between NEN arising at different sites.
- Lung: Studies on the separation between typical carcinoid and atypical carcinoid, and between these entities, SCLC and LCNEC using molecular, histological and protein expression methods. Does a G3 category of lung NET exist comparable to that in the pancreas?
- Pituitary: Studies of the genetics of NET (adenoma/NET, aggressive NET, and carcinoma) are required. It is as yet uncertain if NEC exist at this site.
- Metastases: What are the optimal diagnostic criteria and terminology to be used for metastatic rather than primary NEN?

## Conclusions

We have provided a framework for a common classification of NENs. Morphology is the primary basis for this classification, supported in some sites by underlying genomic alterations. We believe this conceptual approach can form the basis for the next generation of NET classifications and will allow more consistent taxonomy to understand how neoplasms from different organ systems inter-relate genetically. We also recognize the site-specific differences among NENs, which are also of critical importance in their proper diagnosis and clinical management.
